# Recent Advancements in Glaucoma Surgery—A Review

**DOI:** 10.3390/bioengineering10091096

**Published:** 2023-09-19

**Authors:** Bryan Chin Hou Ang, Sheng Yang Lim, Bjorn Kaijun Betzler, Hon Jen Wong, Michael W. Stewart, Syril Dorairaj

**Affiliations:** 1 Department of Ophthalmology, National Healthcare Group Eye Institute, Tan Tock Seng Hospital, Singapore 308433, Singapore; 2 Department of Ophthalmology, National Healthcare Group Eye Institute, Woodlands Health Campus, Singapore 737628, Singapore; 3 Department of Surgery, Tan Tock Seng Hospital, National Healthcare Group, Singapore 308433, Singapore; 4 Yong Loo Lin School of Medicine, National University of Singapore, Singapore 119077, Singapore; 5 Department of Ophthalmology, Mayo Clinic, Jacksonville, FL 32224, USA; stewart.michael@mayo.edu (M.W.S.);

**Keywords:** glaucoma, trabeculectomy, glaucoma tube shunts, minimally invasive glaucoma surgery, device, eye

## Abstract

Surgery has long been an important treatment for limiting optic nerve damage and minimising visual loss in patients with glaucoma. Numerous improvements, modifications, and innovations in glaucoma surgery over recent decades have improved surgical safety, and have led to earlier and more frequent surgical intervention in glaucoma patients at risk of vision loss. This review summarises the latest advancements in trabeculectomy surgery, glaucoma drainage device (GDD) implantation, and minimally invasive glaucoma surgery (MIGS). A comprehensive search of MEDLINE, EMBASE, and CENTRAL databases, alongside subsequent hand searches—limited to the past 10 years for trabeculectomy and GDDs, and the past 5 years for MIGS—yielded 2283 results, 58 of which were included in the final review (8 trabeculectomy, 27 GDD, and 23 MIGS). Advancements in trabeculectomy are described in terms of adjunctive incisions, Tenon’s layer management, and novel suturing techniques. Advancements in GDD implantation pertain to modifications of surgical techniques and devices, novel methods to deal with postoperative complications and surgical failure, and the invention of new GDDs. Finally, the popularity of MIGS has recently promoted modifications to current surgical techniques and the development of novel MIGS devices.

## 1. Introduction

Glaucoma is the leading cause of irreversible blindness worldwide, with little variability according to race, ethnicity, or location [1]. Elevated intraocular pressure (IOP) remains the primary modifiable risk factor for glaucoma progression, thereby mandating that treatments lower the IOP. This is the only therapeutic strategy that prevents damage to the optic nerve and the progression of visual field defects [2]. Anti-glaucoma medications, most of which work by lowering aqueous production or increasing outflow, as well as laser procedures, such as peripheral iridotomy or trabeculoplasty, are generally considered to be first-line therapy [3]. Surgery is usually indicated when glaucoma medications and lasers are unable to reduce IOP sufficiently to halt visual field loss [4].

Trabeculectomy has long been the gold standard for the surgical management of glaucoma, but new surgical techniques and devices, including glaucoma drainage devices (GDD) and minimally invasive glaucoma surgery (MIGS), have been recently developed [5]. Because the field of glaucoma surgery has changed so significantly over the past decade, the authors believe a comprehensive review that consolidates and summarises recent developments and innovations in trabeculectomy surgery, GDDs, and MIGS is warranted.

## 2. Materials and Methods

A comprehensive search of PubMed, EMBASE, and the Cochrane Central Register of Controlled Trials (CENTRAL) was performed on 29th August 2022. Combinations of the following keywords and MeSH terms were used: “Glaucoma”, “Trabeculectomy”, “Glaucoma Drainage Implants”, “Tube”, “Tube Shunt”, “Tube Shunts”, “Ahmed”, “Baerveldt”, “Clearpath”, “Molteno”, “Paul”, “Minimally Invasive Surgical Procedures”, “MIGS”, “Minimally Invasive Glaucoma Surgery”, “Trabectome”, “Trabeculectomy”, “GATT”, “Gonioscopy-assisted transluminal trabeculotomy”, “Trab360”, “iStent”, “Hydrus”, “XEN”, “Preserflo”, “Canaloplasty”, “ABiC”, “iTrack”, “Kahook”, “KDB”, “Omni”, “Visco360”, “Visiplate”, “Cypass”, and “Durysta”. The search was restricted to only adult studies (>19 years of age) and studies published in English. The literature searches for trabeculectomy and GDDs dated back to 29th August 2012 (10 years) and for MIGS dated from 29th August 2017 (5 years). Identified studies were evaluated and manually searched to identify other eligible studies, which were added as hand searches.

Advancements were defined as developments in the following predefined areas: trabeculectomy—“incisional technique” and “closure technique”; GDDs—“GDD surgical technique”, “Existing GDDs”, and “New GDDs”; MIGS—“MIGS technique”, “combination MIGS”, and “new MIGS devices”. Any surgical advancement or development that fell under the predefined categories and was described within the specified time frame was included. A supplementary manual search was conducted if the included study was deemed not to be the original/first description of the advancement. Criteria for inclusion did not include consideration of the significance or extent of real-life adoption of the particular advancement. Only studies involving United States Food and Drug Administration (FDA) or European Conformité Européenne (CE)-approved MIGS devices were included for final review under the MIGS section.

Manuscripts were assessed by three reviewers (S.Y.L., B.K.B., and H.J.W.) for inclusion. Disagreements were resolved through discussion and consensus, and when unsuccessful, a senior reviewer (B.C.H.A.) was consulted.

**Figure 1:**
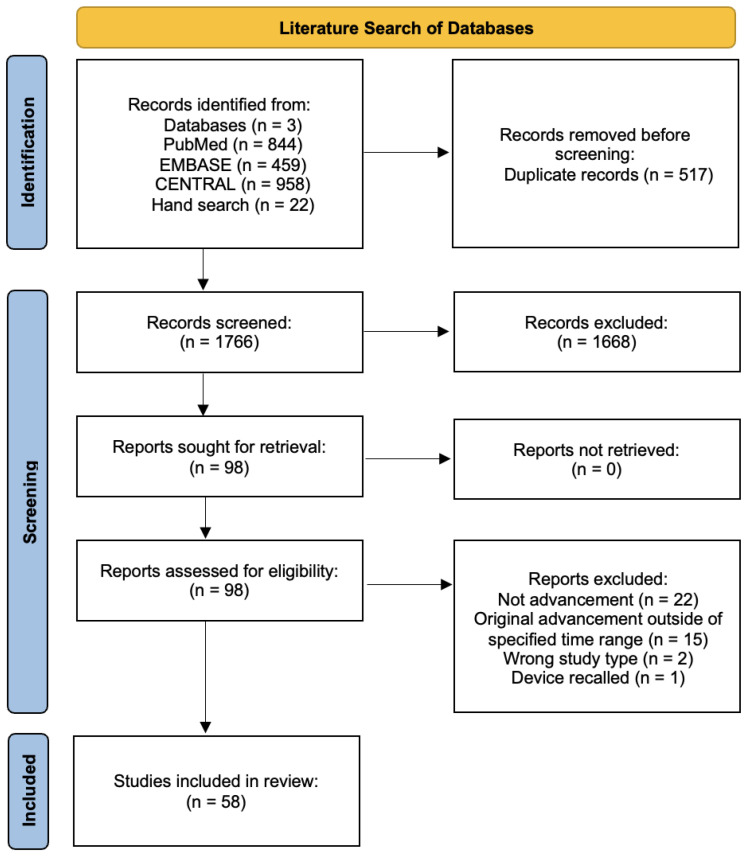
PRISMA flowchart [6].

The database searches yielded 2261 results—844 (PubMed), 459 (EMBASE), and 958 (CENTRAL). An additional 6 (Trabeculectomy), 14 (GDD), and 2 (MIGS) studies were added from hand searches, and 517 duplicates were subsequently removed. After the initial title–abstract sieve, 98 (out of 1766 articles) remained—16 (Trabeculectomy), 38 (GDDs), and 44 (MIGS). Following a full-text review, 58 articles—8 (Trabeculectomy), 27 (GDD), and 23 (MIGS)—were included in the final review on advancements. Results of the literature searches and reviews are presented in a PRISMA flowchart [6] (Figure 1). A summary list of all included studies is presented in Table A1 (Appendix A).

## 3. Trabeculectomy

As a technique to divert aqueous from the anterior chamber into the subconjunctival space, conventional trabeculectomy was first described by Cairns in 1968 [7]. Surgery includes the creation of a fornix or limbal-based conjunctival flap, dissection of the underlying Tenon’s layer, the creation of a partial-thickness scleral flap, the formation of an ostium into the anterior chamber, and finally, a surgical iridectomy to prevent postoperative occlusion of the ostium [8]. Aqueous flows down a pressure gradient from the anterior chamber into the subconjunctival space, resulting in the formation of a filtering bleb with a reduction in the IOP. Trabeculectomy remains the gold standard, first-line, subconjunctival filtration surgery for the treatment of vision-threatening glaucoma.

The performance of an “ideal” trabeculectomy is said to follow the “10-10-10” rule—a surgical time of 10 min, the achievement of a postoperative IOP of 10 mmHg, and an effect that lasts for 10 years or longer [9]. The Moorfields Safer Surgery System, adopted by many trabeculectomy surgeons worldwide, has been designed to facilitate trabeculectomy outcomes following the “10-10-10” rule [10]. Despite the long history of trabeculectomy, challenges, including bleb failure from postoperative fibrosis, concerns regarding long-term IOP-lowering efficacy, bleb complications, such as leaks, hypotony, and endophthalmitis, and the ongoing need for vigilant postoperative monitoring and interventions to sustain surgical efficacy, persist [11]. This section will explore advancements in trabeculectomy surgery that are meant to overcome these challenges and improve outcomes, with a special focus on incisional and closure techniques.

### 3.1. Incisional Technique

Trabeculectomy augmented by limited deep sclerectomy (LDS) was first described in 2017 by Dada et al. [8]. LDS involves elevating and excising a 3 × 3 mm block of deep scleral tissue below the initial scleral flap, thereby creating a crater in the scleral bed [8]. This augmentative intra-operative procedure is intended to surgically thin the remaining sclera, thereby enhancing permeability and increasing aqueous drainage. The pooling of aqueous within the inner scleral layers is intended to promote supraciliary and suprachoroidal outflow of aqueous [8,12] with the size of the pressure difference between the anterior chamber and suprachoroidal spaces driving uveoscleral outflow [8]. These additional filtration pathways reduce reliance on subconjunctival filtration and theoretically allow for greater reductions in IOP. The intrascleral lake supports the scleral flap, preventing its collapse, and scleral flap elevation reduces the risk of local episcleral and intrascleral fibrosis [8]. LDS-augmented trabeculectomy appears to be a potential alternative to conventional trabeculectomy. In a randomised controlled trial of 68 patients with primary open-angle glaucoma or primary angle closure glaucoma with pseudophakia, LDS-augmented trabeculectomy reduced IOP from a baseline of 29 ± 4.6 mmHg to 12.54 ± 1.67 mmHg at 12 months whilst conventional trabeculectomy reduced IOP from a baseline of 30 ± 5.2 mmHg to 13.45 ± 1.83 mmHg at 12 months [8]. None of the eyes in the LDS group required postoperative bleb needling [8], a procedure often performed to revive a non-functional fibrotic bleb. Both LDS-augmented and conventional trabeculectomy reduced the need for postoperative glaucoma medications (3.36 ± 0.48 to 0.46 ± 0.76 vs. 3.34 ± 0.48 to 0.9 ± 0.76) and LDS-augmented trabeculectomy had a lower rate of surgical failure [8].

**Figure 2:**
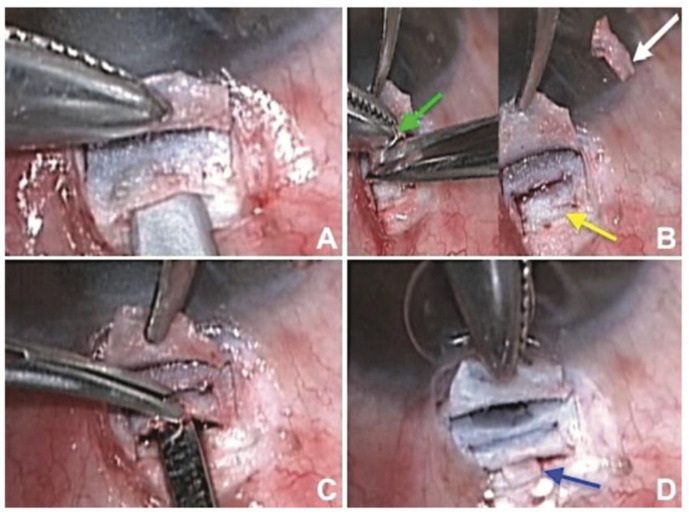
(**A**) The making of a partial-thickness scleral flap to create a deep crater; (**B**) further dissection of the scleral block to create an even deeper crater; (**C**) cyclodialysis cleft made using cyclodialysis spatula; and (**D**) deep scleral tissue inserted at the cyclodialysis cleft. Courtesy of Dada et al. [13].

In 2022, Dada et al. [13] further enhanced the LDS-augmented trabeculectomy by creating a cyclodialysis in two patients who had high IOP after vitrectomy. In this surgical modification, a controlled separation of the ciliary body from the scleral spur is performed at the site of the scleral flap. The excised deep scleral tissue during LDS is used as a spacer in the cyclodialysis cleft (Figure 2) [13], which prevents closure and fibrosis of the cleft, and ensures suprachoroidal drainage of aqueous [13]. The spacer also inhibits the excessive aqueous outflow that commonly occurs with standard cyclodialysis procedures. These alternative drainage pathways alleviate the IOP-lowering burden on the subconjunctival bleb and minimise the risk of bleb-related complications, such as fibrosis and bleb leak. Good short-term IOP outcomes, from 38 mmHg to 12 mmHg and 44 mmHg to 10 mmHg, were seen in both patients, without leaking blebs at 6 months [13]. Unfortunately, the long-term efficacy and safety of this procedure have yet to be reported.

The Extended Subscleral Technique (ESST) was first described in 2014 by Saeed et al. [14] as an adjunct to trabeculectomy. In ESST, a narrow longitudinal strip of deep sclera posterior to the scleral flap is removed to create an extended scleral tunnel, approximately 6 mm in length from the limbus, that allows aqueous passage into the posterior subconjunctival space. The pressure gradient between the original filtering ostium and the additional subscleral tunnel produces a regulated posterior flow [14,15]. Consistent with Bernoulli’s principle, the different channel diameters produce variable aqueous velocities, with regions of low and high pressures. The resultant force balances out the pressure difference, thereby encouraging a posteriorly directed, controlled flow of aqueous. By acting as another outlet for aqueous, the ESST limits aqueous outflow velocity, minimises the development of a shallow anterior chamber post-operatively, and promotes the formation of a more widely-distributed posterior bleb [15]. The enhanced diffusion of aqueous into the wider adjacent subconjunctival space may inhibit the formation of a ring of scar tissue—the “Ring of Steel”—that often forms at the junction between bleb and normal conjunctiva, leading to a localised, elevated, and thin bleb that increases the risk of postoperative leaks [15]. ESST may reduce the need for postoperative bleb needling, a procedure that is often used to re-establish drainage after fibrosis and encapsulation [16]. A randomised controlled trial that examined outcomes of ESST-augmented trabeculectomy vs. conventional trabeculectomy in 40 eyes with primary open angle glaucoma found no bleb-related complications in the ESST group. ESST also produced a greater reduction in IOP from baseline compared to that following conventional trabeculectomy with a superior reduction in IOP that was statistically significant at 7 days (80.0% vs. 56.0%) and 180 days (66% vs. 53.6%), but non-significant at 1 day (66.7% vs. 57.2%) and 1 year post-operatively (67.5% vs. 53.1%) [15]. A statistically greater reduction in the need for postoperative glaucoma medications was observed in the ESST group (2.53 ± 0.9 to 0.052 ± 0.2) compared to conventional trabeculectomy (2.85 ± 0.59 to 0.65 ± 0.2) at 1 year [15].

### 3.2. Closure Technique

Trabeculectomy surgery concludes with suturing of the scleral flap, adjustment of suture tension, and watertight closure of the conjunctiva and Tenon’s layers. These steps are critical for preventing bleb leaks and ensuring adequate postoperative IOP reduction. In recent years, new closure techniques have been developed to improve both the efficacy and safety profile of trabeculectomy.

Chan et al. [17] reported a novel, modified conjunctival closure technique that repositions and separates Tenon’s layer from the conjunctiva, which differs from the traditional simultaneous closure of conjunctiva and the Tenon. The Tenon is dissected from the conjunctiva and anchored close to and overlying the scleral flap; the conjunctiva is then closed separately [17]. This technique positions the inner surface of Tenon’s layer further from the anterior sclera, which lowers the risk of postoperative fibrosis and enhances aqueous flow into the sub-Tenon’s space alongside the intentional misalignment of the Tenon and sclera. This helps maintain space patency and encourages posterior aqueous flow. Approximating the Tenon on top of the partial-thickness scleral flap creates a tensional force that may prevent over-drainage, thereby reducing the risk of hypotony. Anchoring the Tenon allows conjunctival closure with minimal tension, thereby reducing the risk of a buttonhole, dehiscence, and bleb leak. Finally, this closure method reduces the risk of Tenon’s layer retraction and encourages the development of a thicker bleb wall, which may reduce the risk of a cystic bleb and bleb leaks [17]. In this non-comparative case series, 30 Chinese patients underwent fornix-based trabeculectomy with mitomycin C and experienced a reduction in mean IOP from 28.5 ± 9.6 mmHg to 15.5 ± 2.6 mmHg and a decrease in the need for postoperative glaucoma medications (4.4 ± 0.9 to 0.8 ± 0.12). No wound leaks were observed.

The literature describes better long-term results with fornix-based trabeculectomy than with limbal-based trabeculectomy but with greater risks of conjunctival wound leakage [18]. In 2015, Olawoye et al. described a new closure technique for fornix-based trabeculectomy that uses a horizontal conjunctival suture [19]. This ensures a watertight limbal conjunctival wound and mechanically separates the conjunctiva from the cornea. A non-comparative case series of 79 eyes with primary open angle glaucoma or secondary glaucoma, such as exfoliative and pigmentary glaucoma, that were at high risk of surgical failure reported low rates of bleb leakage (7 (8.8%) eyes), as compared to other forms of previously described conjunctival closure techniques in fornix-based trabeculectomy. Significant reductions in mean IOP (31.5 ± 8.1 mmHg to 14.2 ± 6.0 mmHg) and postoperative glaucoma medications (3.7 ± 0.8 to 0.6 ± 0.12) were also observed [19].

Kirk et al. in 2014 [18] modified the original Wise closure technique [20] by creating a limbal lip of conjunctiva. The firm adhesion between the anterior and posterior conjunctival edges promotes healing and minimises leakage at the wound site. The closing suture, which is secured to both the conjunctiva and sclera peripheral to the original conjunctival incision, evenly distributes mechanical traction across the wound [18]. This contrasts with traditional closure techniques that rely heavily on the sutures at both ends of the conjunctival flap [18,21]. A retrospective comparative study (313 patients) that investigated the efficacy and safety profile of the modified Wise closure [21] found that the incidence of bleb leaks was lower after the modified Wise closure than with winged sutures (6.4% vs. 16.6%). The modified Wise closure exhibited a stronger protective effect against bleb leaks compared to techniques employing winged sutures for closure (odds ratio of 0.345; 95% CI 0.16–0.74; *p* = 0.007) [21]. The postoperative IOP reduction from baseline in six months was significantly greater following the modified Wise closure compared to closure with winged sutures (−14.5 ± 10.8 mmHg vs. −11.6 ± 9.1 mmHg) [21] though no significant difference in the need for postoperative glaucoma medications between groups was found.

Figus M et al. first described the use of a scleral flap everting suture for anterior filtering procedures with a scleral flap in 2016 [22]. This technique involved passing an everting 10-0 nylon suture through the distal margin of the flap, then through the limbus twice before knotting and forming a closed ellipse with a loop on the cornea. If IOP reduction is required postoperatively, traction can be applied to the exposed loop to increase aqueous outflow and restore the bleb. In 92 eyes that underwent filtering surgery, the authors reported the need to traction the everting suture by 4 months postoperatively in 26 out of 92 eyes, of which the procedure was successful in reopening the scleral flap in 25 eyes. However, IOP results were not reported, as the abovementioned study was still undergoing approval by the institutional ethics committee. Baykara M. et al. in 2017 reported a modification of the scleral flap everting suture—the accordion suture—in a population of eight eyes with neovascular glaucoma [23]. The technique involved first passing the suture through the mid-distal edge of the scleral flap, internal to external, and then through the mid-left edge of the flap, external to internal. Next, the suture is passed through the clear cornea at the limbus and again through the clear cornea, creating a U-shaped loop. Subsequently, the suture is passed through the mid-right edge of the scleral flap, internal to external, and finally through the mid-distal edge of the flap, external to internal. Lastly, both ends of the suture are placed underneath the scleral flap and finely tied with a 3-1-1 slip knot manner after adjustment of desired tension. The use of the accordion suture has been postulated by the authors to result in an even lifting pressure applied to both edges of the flap, which delivers a more substantive decrease in IOP. The mean removal time of the accordion suture was reported to be 3.5 ± 0 weeks post-operatively, with the mean IOP before and after the procedure at 22.63 ± 2.06 mmHg and 11.12 ± 2.64 mmHg, respectively.

## 4. Glaucoma Drainage Devices

Glaucoma drainage devices (GDDs) have become a mainstay in the surgical management of advanced, refractory glaucoma, particularly in those eyes with a prior history of failed filtering surgery. GDDs divert aqueous humour (AH) from the anterior chamber to an external reservoir, over which a fibrous capsule forms at 4–6 weeks after surgery. AH diffuses between the collagenous fibres of the capsule and is absorbed by capillaries and lymphatic vessels within the Tenon and conjunctiva. The base plate prevents conjunctival adhesion to the sclera and maintains the AH reservoir [24], though the fibrous capsule encapsulating the base plate is the site most resistant to AH flow [24,25]. Overall, GDDs successfully control IOP in eyes with previously failed trabeculectomy [26], and in eyes with prior conjunctiva scarring that precludes other forms of subconjunctival filtering surgery. GDDs are usually classified as flow-restrictive (valved) or non-flow-restrictive (non-valved) types, with devices varying according to size and base plate material. Since the Molteno drainage implant device [27] was first introduced into clinical practice, attempts have been made to improve the safety and efficacy of GDDs by modifying intra-operative techniques, exploring ways to manage postoperative complications and surgical failure, modifying existing devices and creating new GDDs.

### 4.1. Modifications to Existing Techniques of GDD Implantation

Assessing the function and patency of GDDs is important for ensuring good and predictable outcomes after GDD implantation. Grover et al. [28] used Trypan blue to assess adequate GDD flow in three situations: (1) when completing the second stage of Baerveldt tube implantation; (2) when blockage of a valved implant is suspected after it had previously functioned well; and (3) when the valve mechanism of an implant seems to have failed. In the first situation, Trypan blue injection through the drainage tube stained the capsule, signifying that the dye successfully reached the plate. In the second situation, elevated IOP was observed in the first postoperative week after Ahmed Glaucoma Valve (AGV) implantation. To assess for GDD function, diluted Trypan blue was flushed into the tube with blue staining of the capsule and plate, confirming GDD function. In the third situation, high IOP occurred during the second postoperative week after the implantation of an AGV. After initial irrigation attempts had failed, the tube was externalised and flushed aggressively with diluted Trypan blue, thereby re-establishing good flow. In this case, the dye served both to confirm and re-establish flow in the GDD, but the authors also advised against the overuse of Trypan blue because of its association with endothelial toxicity at high concentrations and prolonged exposure [29]. In this study, the authors minimised the risk of toxicity by diluting three drops of the dye with 3 mL of balanced salt solution.

Another modification to the GDD implantation procedure involves the placement of the GDD tube through a sclerotomy port during vitreoretinal surgery. In eyes with compromised anterior segments due to previous surgeries or disease processes, as well as in post-corneal transplant eyes, GDDs have been implanted in the sulcus or vitreous cavity. Gupta et al. [30] described pars plana placement of the AGV through a sclerostomy port in the only-seeing eye of an aphakic patient with post-penetrating keratoplasty refractory glaucoma and a history of trabeculectomy. In this case, the AGV tube was trimmed to an intravitreal length of 6mm and inserted through a superotemporal 25 G vitrectomy port to minimise the number of entry wounds and, hopefully, to limit postoperative fibro-vascular proliferation and exaggerated wound healing [31].

While GDDs usually drain into the subconjunctival space, Maldonado-Junyent et al. [32] followed the principles of a ventriculoperitoneal shunt used in the treatment of hydrocephalus, to drain aqueous humour into the peritoneal cavity. A hydrocephalus valve (Medtronic PS Medical Strata NSC) was used, regulated at level 2.5 to operate at pressures between 14 and 16 mmHg. Good IOP was maintained for the first four weeks, but longer-term results have yet to be published. This unique modification to GDD implantation raises the possibility of diverting aqueous to other spaces outside of the eye.

### 4.2. Novel Techniques to Manage Surgical Complications and Failure following GDD Implantation

Tube exposure is a well-known complication of GDD implantation [33] that may result from the eye’s immunologic response, repeated mechanical irritation caused by blinking, outward pressure against the tube from the eye, or vaulting of the tube due to intrinsic tube elasticity. Various strategies, including the creation of an overlying scleral flap and the application of patch grafts, have been used during surgery to reduce the incidence. In a retrospective series of 36 eyes with refractory glaucoma, Ma et al. [34] used a modified scleral tunnel technique. After the tube was inserted into the scleral tunnel, it was covered by both the tunnel and an overlying scleral flap at the point of intersections. Through 21 months of follow-up (mean), no conjunctival tube exposure was reported.

**Figure 3:**
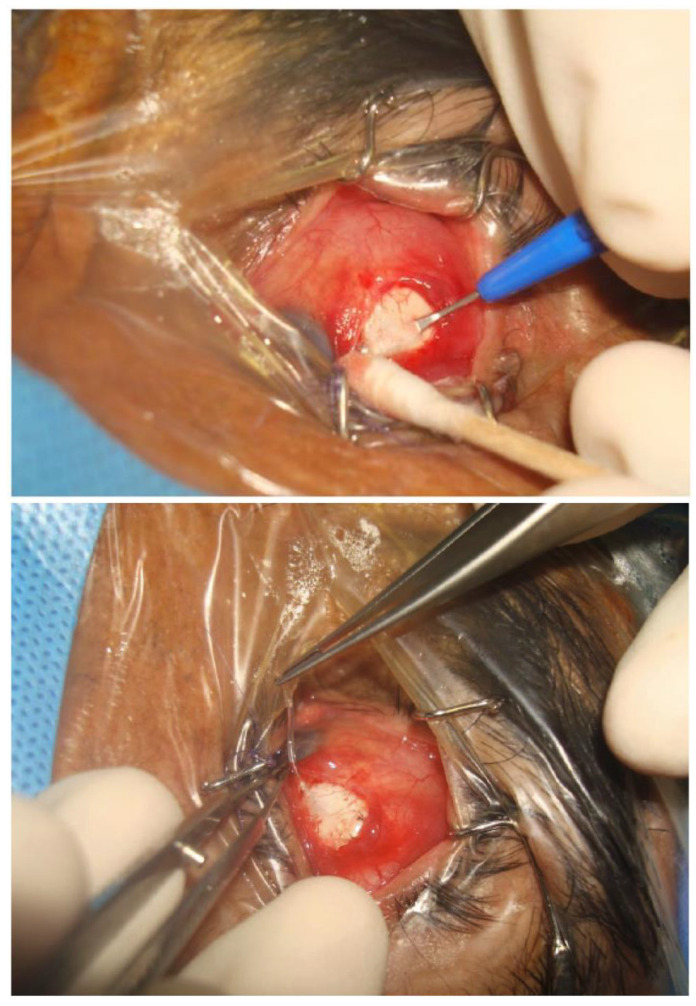
Creation of an 8 mm half-thickness scleral tunnel (**top**); passage of shunt tube through the scleral tunnel (**bottom**). Courtesy of Eslami et al. [35].

In a retrospective series of 30 eyes, Eslami et al. [35] reported the use of a single long tunnel to prevent tube exposure. The authors proposed that preventing tube-conjunctiva contact would reduce the risk of tube exposure, and through a 37.2-month follow-up (mean), no cases of tube exposure were reported. The surgical technique (Figure 3) begins with an 8 mm half-thickness scleral tunnel, after which the plate of the shunt device is secured to the sclera. The silicone tube is trimmed, threaded through the scleral tunnel, and inserted into the anterior chamber under a scleral flap and through a partial paracentesis. The limbal scleral flap is closed to prevent leakage.

**Figure 4:**
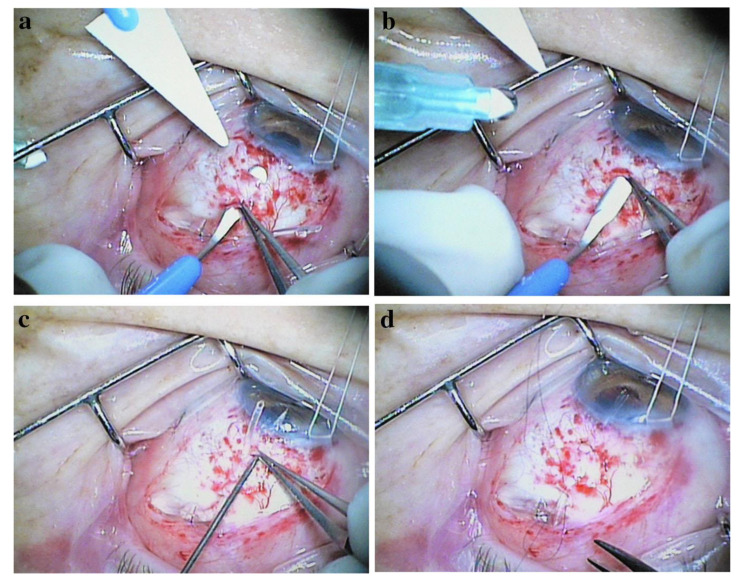
(**a**) The distal-to-limbus tunnel is prepared with a bevel-up lancet between the two scleral incisions; (**b**) the proximal-to-limbus tunnel is fashioned from the proximal-to-limbus incisions to the limbus; (**c**) a paracentesis is created with a 23-gauge needle through the proximal-to-limbus tunnel into the anterior chamber; (**d**) the tube is secured with a 10-0 nylon suture (distal incision-sclera, sclera-distal incision). Courtesy of Brouzas et al. [36].

Brouzas et al. [36] developed a ‘double scleral tunnel in tandem’ technique to exceed the maximum length of a single tunnel. Two scleral incisions are made parallel to the limbus at 4 and 12 mm. A half-thickness scleral tunnel is dissected between the two incisions (Figure 4), and a second tunnel is made from the proximal incision to the limbus. After injection of a viscoelastic into the anterior chamber and the creation of a paracentesis, the tube is inserted through both the distal and proximal tunnels, and then into the anterior chamber. The proximal incisions are sutured, and the tube and conjunctiva are secured. In a series of 28 eyes, only two (7.1%) cases had tube exposure after a mean follow-up of 60 months.

**Figure 5:**
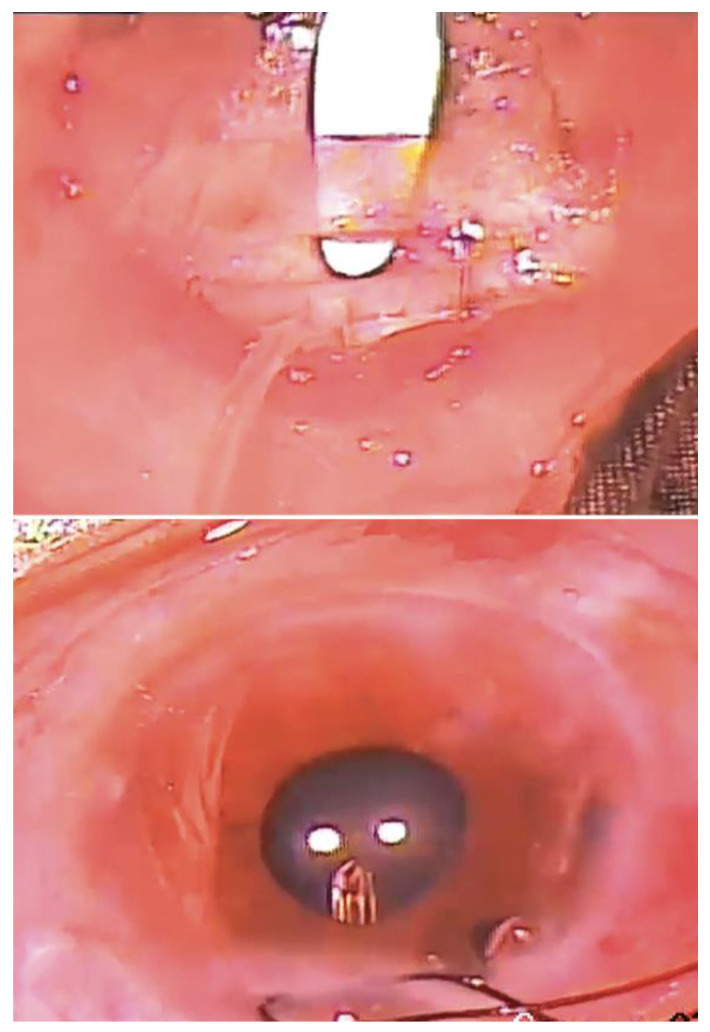
(**top**) The lamellar scleral tunnel was created with a crescent blade for the passage of the AGV tube. (**bottom**) Insertion of the tube in the sulcus. Courtesy of Prakavan et al. [37].

Alternative techniques to cover the GDD implant during surgery have also been described. In a randomised clinical trial, Pakravan et al. [37] reported the use of a graft-free, short tunnel, small flap method of AGV implantation and compared it with a scleral patch graft. Comparable success rates, including postoperative IOPs, glaucoma medication burden, and complication rates through 1 year, were found with each approach. These data suggest that the graft-free, short tunnel, small flap technique may be a viable way to reduce the risks associated with scleral patch grafts. Gupta et al. [38] also used a graft-free scleral sleeve technique (Figure 5) in a single patient during the COVID-19 pandemic to reduce the risk of viral transmission through a donor scleral graft and reported no tube exposure through 6 months.

GDD efficacy is often limited by bleb fibrosis, with no clear consensus on the effectiveness of intra-operative antimetabolite use in reducing the rate of surgical failure [39]. Alternate adjuncts have been used with the hope of preventing bleb fibrosis following GDD implantation. In a randomised prospective multicentre clinical trial with 58 patients, Sastre-Ibanez et al. [40] used the Ologen collagen matrix, but could not demonstrate an efficacy or safety benefit over traditional AGV implantation surgery after 12 months postoperatively.

New surgical techniques have been developed to better manage intra-operative complications. Mungale et al. [41] described a novel method to manage inadvertent tube-cut by a ligature that sometimes occurs during aurolab aqueous drainage (AADI) implant surgery. The authors removed the short end of the tube attached to the implant and reinserted the long, transected end into the back plate of the implant. Management options, in this case, were limited by the absence of spares and other materials for tube extension, and the authors cautioned that similar techniques might not be applicable to valved implants like the AGV, where the tube fits tightly into the base plate.

Early GDD failure may occur because a blood clot obstructs the tube, particularly in eyes with neovascular or inflammatory glaucoma. At the end of surgery, Hwang et al. [42] injected filtered air into the anterior chamber through a 30-gauge needle. The authors hypothesised that a large air bubble would keep blood from entering the tube opening and prevent an obstructive clot from forming.

GDDs may also become occluded by iris tissue. In a single case, Kataria et al. [43] used a single trans-corneal suture to manage iris tuck in an AADI tube. A trans-corneal sling suture was passed through the cornea and behind the tube, approximately 2 mm from the limbus. The suture tension was adjusted to lift the tube away from the iris while keeping it a safe distance from the corneal endothelium. The authors acknowledged, however, the risks of suture-related infection, corneal astigmatism, and persistent tube-iris or tube-cornea touch.

Surgical options, including the implantation of additional GDDs and “piggyback” drainage devices, have been developed to treat primary GDD failure [44]. In a series of 8 eyes, Lee et al. [45] reported the implantation of an additional AGV device in patients with IOP persistently ≥30 mmHg, despite having a GDD and receiving maximally tolerated medical therapy. Seven (of eight) patients had a statistically significant decrease in glaucoma medications 1 year post-operatively. No cases of diplopia or corneal decompensation were observed. In 16 eyes of 14 patients with uncontrolled glaucoma, Valimaki et al. [46] inserted a second glaucoma drainage implant in a piggyback manner. The sequential implant was rotated so that the tube of the ‘piggyback’ implant was directed towards the quadrant containing the original implant and inserted into the bleb, thereby converting a one-plate into a two-plate implant. The mean IOP was reduced from 29.2 mmHg to 17.3 mmHg, suggesting that a piggyback approach may be a viable option in patients with a failed GDD. In a series of 18 eyes, Dervan et al. [47] sutured a Baerveldt (250 or 350 mm) or Molteno3 GDD into an unused scleral quadrant and connected the silicone tube to the primary plate bleb. Mean IOP was reduced from 27.1 mmHg to 18.4 mmHg at the last follow-up. Several studies [44,48,49] suggest that piggyback GDD placement may be a viable surgical option for primary tube failure, without risking the corneal decompensation that may occur when inserting a second GDD into the anterior chamber [45,46].

Tube retraction, a complication of GDD implantation, often requires surgical revision to maintain drainage. Chiang et al. [50] reported successful outcomes in three patients with a ‘tube-in-tube’ technique that extended the existing tube of the Baerveldt GDD. The anterior portion of the drainage tube was exposed, and its patency was assessed. A tube segment from either a new GDD or a Tube Extender was inserted into the original tube, or vice versa. Advantages of this technique include the need for only minimal surgical dissection and disruption of the pre-existing GDD bleb, having a low risk of joined tube migration due to the high tensile strength, not requiring fixation sutures at the ‘tube-in-tube’ interface, not requiring additional scleral grafting, and the ease with which this technique can be learned. No tube migration occurred during follow-up periods of 1 month to 3 years.

The EX-PRESS Glaucoma Filtration Device (Alcon Laboratories, Fort Worth, TX, USA) has also been implanted in different locations when required by a unique clinical situation. Yen et al. [51] described an eye that had previously undergone pars plana vitrectomy (PPV) with prolonged silicone oil tamponade (22 months) for a rhegmatogenous retinal detachment and had developed neovascular glaucoma (NVG). Trabeculectomy with EX-PRESS implantation was performed, but bleb failure developed three times in four years, and the IOP reached 40 mmHg despite topical anti-glaucoma medications and oral acetazolamide. The existing EX-PRESS device was re-implanted into the posterior segment, and the IOP remained at 8 mmHg for more than 8 months after surgery and without medications [51].

### 4.3. Modifications to Existing GDDs

Over the past few years, GDDs have been repeatedly modified to enhance safety and efficacy. In 42 patients with neovascular glaucoma, Gil-Carrasco et al. [52] compared the safety and efficacy of the AGV model M4 (high-density porous polyethylene plate) and the model S2 (polypropylene plate). The AGV model M4, because of its porous polyethylene plate, was believed to increase aqueous outflow, but no differences in efficacy were seen at 1 year.

### 4.4. Invention of New GDDs

The Paul Glaucoma Implant (PGI) was created to reduce complications while preserving efficacy [53]. The PGI differs by having a smaller tube diameter—the external tube diameter is 467 μm, and the internal tube diameter is 127 μm. By occupying less space in the anterior chamber and preserving a large endplate surface area for aqueous absorption, damage to the corneal endothelium and risk of tube erosion are theoretically lowered [53]. The smaller tube calibre makes intraoperative surgical occlusion easier. At 24 months [54], complete success was achieved in 71.1% of patients, and the mean number of glaucoma medications decreased from 3.2 to 0.29. Complications included a self-limiting shallow anterior chamber, hypotony that required intervention, and tube occlusion.

The Ahmed ClearPath GDD (ACP, New World Medical, Rancho Cucamonga, CA, USA) [55] was introduced in 2019 as a valveless device, available in both 250 and 350 mm^2^ sizes, and with a flexible plate that conforms to the curvature of the globe. Anteriorly located suture fixation points make implantation easier, the posteriorly positioned plate on the 350 model avoids muscle insertions, and an optional pre-threaded 4-0 polypropylene rip cord and a co-packaged 23-gauge needle simplify the creation of a sclerostomy. The lower profile of the plate purportedly reduces the risk of conjunctival erosion and produces a low, diffuse bleb [56]. In a multicentre retrospective analysis of 104 eyes with medically and/or surgically uncontrolled glaucoma, Grover et al. [55] reported good IOP outcomes with both the 250 or 350 mm^2^ devices. Significant reductions in mean IOP (13.6 to 16.7 mmHg) and medications (3.9 to 1.9) were seen at 6 months [55].

A series of newly designed GDDs can be adjusted post-operatively to reduce the incidences of hypotony and hypertension. These devices include the eyeWatch (eW, Rheon Medical, Lausanne, Switzerland) and others currently undergoing animal testing [57,58]. The eW has a deformable silicone tube that can undergo targeted compression to alter its cross-sectional area and thereby change fluidic resistance. Post-operatively, IOP may be changed non-invasively by moving the position of an internal magnetic rotor with an external control unit (the “eyeWatch Pen”). A pilot study found fewer postoperative episodes of hypotony and IOP spikes, with a complete success rate of 40% [59]. Subsequent studies produced outcomes comparable to those with the AGV [60]. Adjustable GDDs may reduce the need for intra-operative measures, such as tube ligation, and enable better postoperative IOP control.

The primary objective of GDDs has been to improve the drainage of aqueous, but GDDs are now being developed as extended-release drug reservoirs [61,62]. No US FDA-approved GDD drug delivery systems have reached the market, but base plates are being redesigned as reservoirs for drug storage. The tube would deliver the drug into either the anterior or posterior chamber through a one-way pressure-dependent valve. A wireless programming system is being developed to control drug delivery [63,64] with challenges that include the creation of a micro-delivery system and the need to resupply the reservoir.

**Figure 6:**
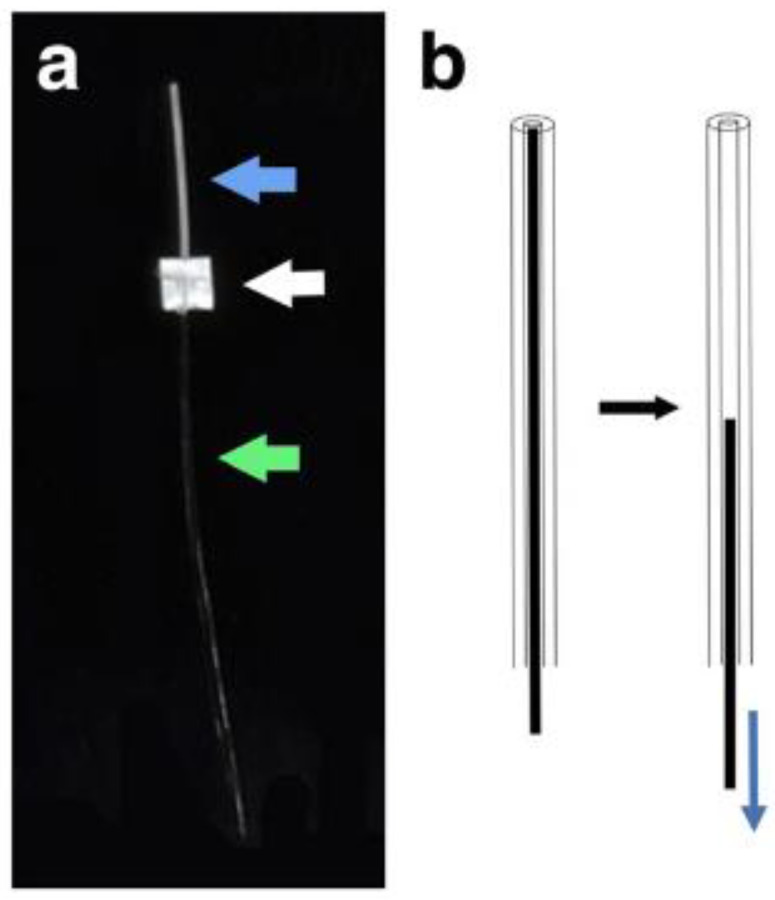
(**a**) The MicroMT consists of an expanded polytetrafluoroethylene membrane (white arrow) and silicone tube (blue arrow) with an intraluminal stent (green arrow); (**b**) the stent can be retracted after the operation. Courtesy of Ahn et al. [65].

Base plates may be replaced with tube shunt devices that have expanded membranes. In a study of 43 eyes, Ahn et al. [65] reported that the MicroMT (Figure 6), a membrane-tube shunt device, significantly reduced IOP from 22.5 mmHg to 11.1 mmHg after 3 years. The MicroMT has a reduced device profile, which decreases the risk of diplopia and conjunctival erosion.

## 5. Minimally Invasive Glaucoma Surgery (MIGS)

Minimally Invasive Glaucoma Surgery (MIGS) refers to a group of IOP-lowering surgical procedures that have emerged during the last decade. MIGS generally cause minimal trauma with little or no scleral dissection or conjunctival manipulation [66], incorporate either an ab interno or ab externo approach, and have good safety profiles and rapid recovery times [66]. MIGS are broadly classified into the following three categories according to the site of implantation or augmentation [67]: (1) angle-based MIGS, which enhance trabecular outflow by bypassing or manipulating angle structures, such as the trabecular meshwork and Schlemm’s canal; (2) suprachoroidal MIGS, which increases uveoscleral outflow through a suprachoroidal drainage shunt; and (3) subconjunctival MIGS, which creates an aqueous outflow pathway into the subconjunctival or sub-Tenon’s space.

MIGS procedures have evolved rapidly over the past decade, with continuing, robust research and development into new techniques and devices [68]. As with any recently developed surgical device or technique, various challenges have emerged in the performance of surgery and the management of complications. Many of these challenges are common to all MIGS procedures, and they can be broadly classified as follows: (1) perioperative challenges (e.g., difficulty with intra-operative handling, visualisation, and implantation of the device, or bleeding and hypotony in the immediate postoperative period); and (2) long-term postoperative problems (e.g., bleb fibrosis, scarring, stent occlusion, and insufficient long-term IOP lowering). In general, these perioperative and long-term postoperative problems tend to be mild [69,70], and serious sight-threatening complications, such as retinal detachment or endophthalmitis following MIGS, are rare [69,70]. Areas for improvement remain, and since MIGS are becoming an increasingly important option for the management of glaucoma, they are being continuously evaluated [5].

Recent advancements in MIGS have attempted to address current limitations in surgical success rates and ease of use in the following ways: (1) modifications to existing MIGS techniques, (2) combination MIGS, and (3) development of new MIGS. The next section will explore recent advancements in MIGS procedures and devices, and provide examples as to how they attempt to address existing limitations.

### 5.1. Recent Modifications to MIGS Techniques

The XEN45 gel stent (Allergan, Dublin, Ireland), a subconjunctival MIGS device, has demonstrated good safety and efficacy in the management of open-angle glaucoma [71], but many investigators have reported the need for postoperative interventions, such as bleb needling, with or without antifibrotic usage, to maintain the long-term patency of the device and sustain its IOP-lowering effect [72,73]. These additional interventions impose additional cost, risk, and inconvenience to both the patient and surgeon. The XEN45 was originally approved by the US FDA to be implanted with an ab interno, closed conjunctiva technique [74], but glaucoma surgeons have adopted an ab externo approach (with either opened or closed conjunctiva) in an attempt to improve safety, efficacy, and ease of implantation [75,76,77]. Some studies have reported higher rates of surgical success and IOP-lowering and lower rates of bleb interventions in the open conjunctiva ab externo approach. A retrospective case series by Tan et al. [75] showed a greater mean IOP reduction in the ab externo open conjunctiva group compared to the ab interno closed conjunctiva group (12.8 ± 3.0 mmHg (40.1% decrease) vs. 8.4 ± 1.7 mmHg (28.6% decrease); *p* = 0.208) at the 12-month follow-up. Needling was required in fewer ab externo than ab interno cases (26.7% vs. 42%; *p* = 0.231), but the superiority of the ab externo open conjunctiva technique has not been consistently demonstrated across studies [75,76,77].

The distal end of the XEN Gel Stent can become obstructed by Tenon’s, so a transconjunctival ab externo implantation approach [78] has been developed to produce a similar lowering of IOP and medication dependency as the ab interno closed conjunctiva approach but with shorter surgical times and quicker postoperative visual recovery [78]. Another technique to improve XEN implantation in the subconjunctival space is the XEN ‘Air’ Technique [79]. Prior to placement of the XEN gel stent, air and viscoelastic is injected into the subconjunctival space to create a mixed pneumatic/viscoelastic dissection, thus preparing a subconjunctival pocket for subsequent XEN insertion with a larger bleb to reduce rates of postoperative fibrosis.

**Figure 7:**
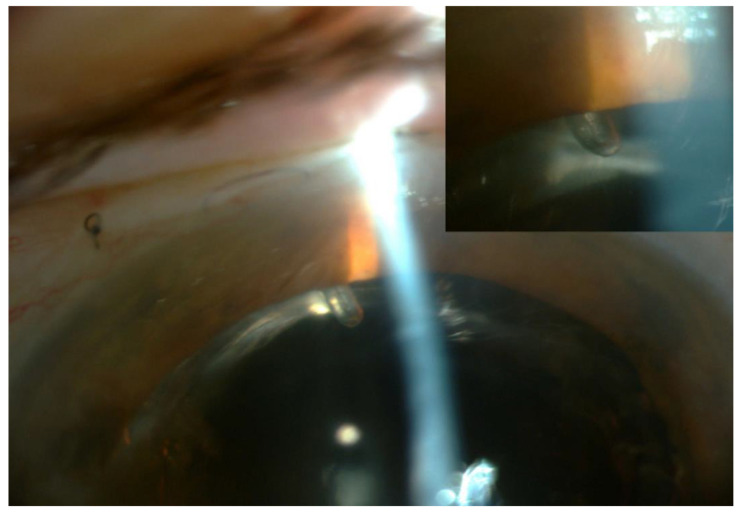
Preserflo Microshunt implanted into the posterior chamber. Note the orientation of the bevel to avoid incarceration of the iris. Courtesy of Martinez-de-la-Casa et al. [81].

The Preserflo Microshunt (Santen, Osaka, Japan) is a similar subconjunctival MIGS device but is meant to be implanted via an ab externo approach into the anterior chamber through an opened conjunctiva. The Preserflo Microshunt has a significantly smaller diameter than other drainage devices, but it still may damage the corneal endothelium [80], particularly if the implant extends far into the anterior chamber or close to the endothelium. Martinez-de-la-Casa et al. [81] reported a patient with open-angle glaucoma refractory to medical therapy (with an IOP of 26 mmHg on maximal medical therapy) and concomitant granular corneal dystrophy with incipient stromal folds and an endothelial count of 700 cells/mm^2^. The Preserflo Microshunt was implanted into the posterior chamber to minimise the possibility of further endothelial damage, and to avoid iris incarceration, the bevel was directed downward as it is when posterior chamber drainage devices are implanted (Figure 7). Six months after surgery, the implant remained functional, with an IOP of 9 mmHg and without additional medical treatment [81].

Poor visualisation may prevent the implantation of MIGS devices. Extensive anterior synechiae or significant corneal opacities may prevent visualisation of the angle through conventional gonioscopy, which increases the risk of implantation failure or precludes MIGS usage entirely. To overcome this challenge, glaucoma surgeons have used intraoperative optical coherence tomography (iOCT) [82,83]. Junker et al. [84] reported the use of iOCT to accurately visualise a Trabectome within iridocorneal structures and facilitate the removal of the trabecular meshwork (TM). Ishida et al. [85] used the iOCT to visualise angle structures during ab interno trabeculotomy with the Tanito microhook (M-2215, Inami, Tokyo, Japan). Further research into the outcomes of iOCT-assisted MIGS procedures may improve the overall safety and success of MIGS while enabling patients who were previously ineligible to undergo these surgeries successfully.

Deep learning can create three-dimensional images of iridocorneal structures during angle-based MIGS surgeries to augment direct microscope visualisation [86]. A recent publication [87] by the Artificial Intelligence in Gonioscopy (AIG) Study Group described a convolutional neural network (CNN) that had been trained on videos of gonioscopic ab interno trabeculotomy with the Trabectome to accurately identify the TM in real-time. The CNN developed by Lin et al. [87] managed to consistently identify the TM from surgical videos, outperforming the human experts against which it was tested. Since accurate identification of iridocorneal structures on gonioscopy may be difficult, and errors can lead to surgical complications or suboptimal outcomes, a real-time assistive deep learning model could have applications to MIGS training and intraoperative guidance [87]. Deep learning could also be useful for other MIGS and non-MIGS glaucoma surgeries.

Existing MIGS devices have also been modified to improve device delivery and facilitate surgical handling. The iStent inject (Glaukos Corporation, San Clemente, CA, USA) consists of two trabecular-bypass flange devices designed to facilitate aqueous outflow into Schlemm’s canal by bypassing the trabecular meshwork. Randomised controlled trials [88,89] showed a good lowering of IOP and a substantial reduction in postoperative medication use. Additional improvements resulted in the iStent infinite—consisting of three wider-flange devices (increased from 230 μm to 360 μm) on a single preloaded injector. The widened flanges optimise stent visualisation and improve placement, while possibly reducing the risk of stent occlusion by the iris. Additional iStent devices further lower IOP, with an incremental benefit of 3 stents over 2 [90]. The new iStent infinite allows the surgeon to inject three devices while entering the eye only once, thus reducing surgical time and risk. A 12-month multicentre clinical trial showed that the iStent infinite [91] significantly and safely reduces IOP in patients with uncontrolled open-angle glaucoma. The original iTrack microcatheter circumferentially viscodilates and intubates the Schlemm’s canal [92], whereas the new iTrack Advance utilises the same microcatheter with a new and improved handheld injector to increase predictability and control during device advancement or retraction. This may reduce complications related to inappropriate device handling or insertion.

### 5.2. Combination MIGS Procedures

The different but complementary mechanisms of action of MIGS procedures have been combined to effectively lower IOP. The OMNI surgical system (Sight Sciences Inc., Menlo Park, CA, USA) was US FDA-approved in 2021 [93] to perform both canaloplasty (microcatheterisation and transluminal viscodilation of Schlemm’s Canal) and trabeculotomy (cutting of TM). This procedure targets the three main sites of outflow resistance in the conventional aqueous outflow pathway—the TM, Schlemm’s canal, and the distal collector channels [94]. Three-hundred-and-sixty-degree catheterisation and pressurised viscodilation enlarge Schlemm’s canal and dilate distal collector channels, thereby removing distal blockages to aqueous outflow and reducing distal outflow resistance. By addressing both proximal and distal areas of outflow resistance, the OMNI surgical system has the potential to increase the IOP-lowering efficacy of a single-setting procedure [94,95,96].

MIGS has been used in combination with traditional glaucoma filtering surgery. To mitigate hypotony and corneal endothelial cell loss [97] after placement of the Baerveldt tube (Advanced Medical Optics, Inc., Santa Ana, CA, USA), D’Alessandro et al. [98] placed (ab externo) an XEN implant into the anterior chamber and inserted the Baerveldt tube more posteriorly. The newly formed double tube was sutured and covered by the scleral flap [98]. In eyes with refractory open-angle glaucoma, Bravetti et al. [99] reported a significant IOP decrease from baseline to 12 months (29.9 ± 13.2 to 15.2 ± 6.6 mmHg (−49.2%); *p* < 0.0001) and medication use decreased from 3.0 ± 1.3 to 1.3 ± 0.9. However, 41.5% of patients required revision surgery or transscleral cyclodestruction, ocular hypotony (under 6 mmHg for >4 weeks) occurred in 24.4% of eyes, and blockage of the XEN gel stent occurred in 17.1%; no cases of corneal endothelial damage were reported.

### 5.3. Recent Development of New MIGS

More MIGS devices have been proposed to overcome the limitations of existing devices, provide new mechanisms for aqueous outflow, facilitate ease of use, or improve device efficacy. The following six MIGS devices will be discussed: (1) MINIject DO627 (iStar Medical, Wavre, Belgium); (2) Intra-Scleral Ciliary Sulcus Suprachoroidal Microtube; (3) iDose TR (Glaukos Corporation, California, USA); (4) Beacon Aqueous Microshunt (MicroOptx, Maple Grove, MN, USA); (5) Minimally Invasive Micro Sclerostomy (MIMS; Sanoculis Ltd., Israel); and (6) STREAMLINE^®^ Surgical System (New World Medical, Rancho Cucamonga, CA, USA).

**Figure 8:**
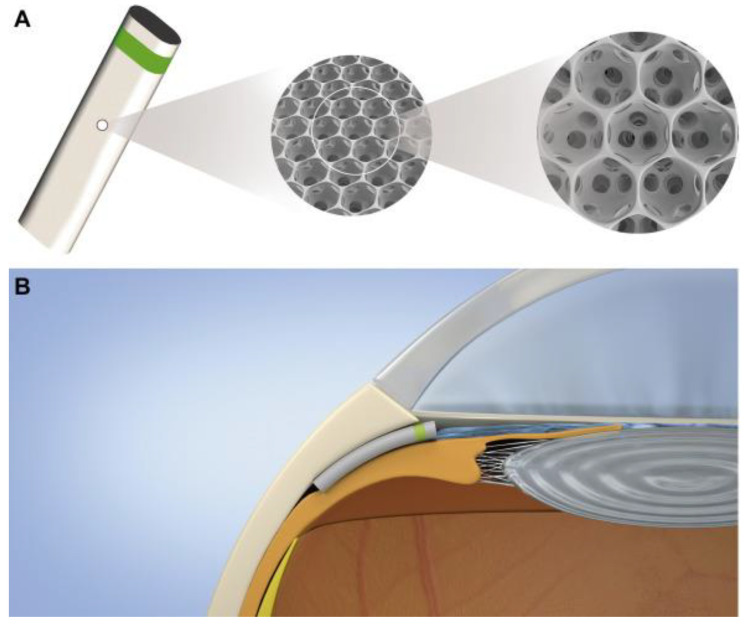
MINIject glaucoma drainage device (iSTAR Medical SA, Wavre, Belgium): (**A**) implant made of STAR material; (**B**) schematic of the device in situ. Courtesy of Denis et al. [100].

The MINIject, a suprachoroidal device inserted ab interno into the supraciliary space [100], has garnered significant interest among glaucomatologists. Compared to angle-based MIGS, supraciliary stents are not limited by downstream episcleral venous pressure, which theoretically allows them to produce greater IOP lowering. Supraciliary stents do not form blebs, thereby eliminating bleb-related risks and interventions, though they are prone to postoperative scarring, tissue reaction, and implant failure [101]. Different suprachoroidal shunts have been introduced over the past decade, with varying degrees of success. Despite initial success, the CyPass Micro-Stent (Alcon Laboratories, Inc., Fort Worth, TX, USA) was withdrawn from the global market in August 2018 due to long-term safety concerns over endothelial cell loss [102]. The SOLX gold shunt (SOLX, Inc., Waltham, MA, USA) did not receive US FDA approval due to high fibrosis-related failure rates [103]. The MINIject DO627 [100] aims to overcome the limitations of previous suprachoroidal MIGS devices by using a biocompatible, medical-grade silicone (STAR material NuSil med-6215) that is soft, flexible, and inherently antifibrotic [104]. The 5 mm long implant does not have a patent lumen but rather consists of a meshwork of porous microspheres that allows aqueous to drain down the pressure gradient at a steady state via a sponge effect (Figure 8). In addition, the silicone demonstrates good biointegration, as surrounding tissue colonises the porous structure while preserving drainage and minimising fibrosis and scarring, thereby eliminating the risk of a blocked lumen [100]. Three clinical trials (STAR-I [100], STAR-II [105], and STAR-III [106]) across 11 sites in Central and South America, Asia, and Europe, showed promising IOP-lowering results and medication reduction over 24 months with few adverse events [107]. The ongoing STAR-V [108] trial aims to enrol 350 patients with primary open-angle glaucoma in the US, and the STAR-VI trial will evaluate the MINIject DO627 in patients undergoing concurrent phacoemulsification.

The ‘Intrascleral Ciliary Sulcus-Suprachoroidal Microtube’ [109] consists of a sterile medical grade silicone tube (Tube extender, New World Medical) with a 300 µm internal diameter and 600 µm external diameter. During insertion, the tube is custom cut, inserted through an inferotemporal conjunctival peritomy to preserve the superior conjunctiva for future surgery, sutured to the sclera to prevent migration, and covered by a partial thickness scleral flap. In a 12-month trial of 36 pseudophakic Black and Afro-Latin patients with glaucoma refractory to topical ocular antihypertensive medications, IOP decreased (21 ± 8.2 to 13.5 ± 4.4 mmHg; *p* = 0.032), as did the mean number of medications (4.2 ± 1.0 to 2.4 ± 1.7; *p* = 0.021), with five patients being medication free. This technique avoids bleb-related complications from traditional trabeculectomy or subconjunctival filtering devices, but there are no data regarding rates of suprachoroidal space scarring or corneal endothelial damage. A larger sample size with longer follow-up is needed.

The iDose TR is a drug-eluting MIGS device that aims to overcome barriers to long-term topical therapy, including patient non-compliance, ocular surface irritation, and difficulty with instilling eye drops [110]. The 1.8 × 0.5 mm biocompatible titanium implant has three main parts—a scleral anchor that affixes to the TM, the body that serves as a reservoir for the drug (travoprost), and a membrane that elutes the drug intracamerally for a target duration of 6–12 months [111]. The iDose TR is implanted similarly to the iStent inject, another MIGS device that is located in the TM. Two phase III randomised controlled trials [112,113] are ongoing, with preliminary results showing that the iDose TR arms will achieve the primary efficacy endpoint of non-inferiority to the active comparator arm (twice-daily topical timolol 0.5%) at 3 months [114]. A favourable safety profile with no clinically significant corneal endothelial cell loss through 12 months was reported [114]. IOP-lowering is likely to diminish after 12 months when the reservoir empties, which will prompt the question of whether the empty implant should be left in place, refilled, or removed.

The Beacon Aqueous Microshunt [115] is a new class of ab externo MIGS that is implanted at the superior limbus to allow aqueous outflow into the tear film. The microshunt measures 1.70 mm wide by 3.30 mm long, with a 0.03 mm × 0.048 mm internal hydrogel channel. Controlled-outflow resistance depends on the channel diameter, and the shunt has been engineered to produce IOP reductions of 8 to 12 mmHg regardless of baseline [115]. To reduce retrograde bacterial movement and mitigate the risk of endophthalmitis, the polyethylene glycol (PEG) hydrogel channel is composed of anti-biofouling polymers that only allow a one-way laminar flow of aqueous humour towards the ocular surface. In a five-patient safety trial [116], no short-term corneal or infectious complications were seen. In a separate, single-patient case report, a significant IOP reduction from baseline (33 mmHg to 12 mmHg) was achieved. Long-term safety and efficacy need to be further investigated [115].

Minimally Invasive Micro Sclerostomy (MIMS) is an ab interno, stent-free, subconjunctival filtration procedure [117]. The MIMS handpiece consists of a 600 µm needle that rotates around its longitudinal axis and has been designed to carve a permanent tunnel near the corneoscleral junction to connect the AC with the subconjunctival space. MIMS is being touted as a MIGS procedure without foreign body-related complications, such as conjunctival erosions, corneal endothelial cell loss, stent migration, or extrusion, while delivering an IOP reduction that resembles existing subconjunctival MIGS. In an early clinical trial with 31 eyes, short-term IOP was lowered, similar to that expected with subconjunctival filtering MIGS [117]. Iris clogging of the internal sclerostomy causing high IOP spikes was the most common and concerning complication, and some of these could not be cleared with laser [117].

The STREAMLINE^®^ Surgical System (New World Medical, Rancho Cucamonga, CA, USA) [118] is a handheld MIGS device for incisional goniotomies and Schlemm’s canal viscodilation. A stainless-steel cannula tip with a retractable outer sleeve is used to make up to eight incisional goniotomies (150 µm diameter each) in the TM, while simultaneously delivering approximately 7 µL of viscoelastic per incision into the Schlemm’s canal. In a series of 19 eyes [118], mean IOP reduction was 8.8 mmHg (36.9%) at 6 months, 57.9% (11/19) of subjects were using fewer medications than at screening, and 42.1% (8/19) were medication-free. A prospective randomised study comparing the safety and efficacy of the STREAMLINE^®^ Surgical System to the iStent inject is ongoing [119].

## 6. Limitations

While this review aims to be a comprehensive one, several limitations are acknowledged. First, to ensure recency of the reviewed surgical procedures and modifications, the scope of this study was limited to trabeculectomy and GDD studies in the last 10 years, and MIGS studies in the last 5 years. Important modifications with significant impact on surgical outcomes may have been introduced outside this timeframe. Second, the emphasis on recent, novel procedures and modifications resulted in the inclusion of case reports and small case studies. This may limit the applicability of this review to the general population. Finally, while this review highlights individual surgeries and procedures, it does not suggest any particular approach to procedure selection in different disease contexts and, hence, may be limited in its clinical applicability.

## 7. Conclusions

There have been significant advancements in all major types of glaucoma surgery—trabeculectomy, GDD implantation, and MIGS. The increasing armamentarium of available surgical procedures and modified techniques will allow glaucoma surgeons to further personalise a patient’s surgical treatment based on the desired magnitude of IOP reduction and anatomical and disease characteristics of the eye, whilst considering the risk-benefit ratio of various techniques. Despite its long history, trabeculectomy surgery continues to be improved with adjunctive incisions, Tenon’s layer positioning, and novel suturing techniques. GDD implantation has also been the subject of several surgical and design modifications. The rapid development of MIGS procedures and their widespread adoption appears to be fuelling further development, including novel modifications to surgical techniques, the development of new MIGS devices, and the emergence of combination MIGS with multiple mechanisms of action to lower IOP.

## Data Availability

This manuscript makes use of publicly available data from published studies. As such, no original data are available for sharing.

## Appendix A

**Table A1:** Summary table of all included studies.

Trabeculectomy Studies		
Author/Year	Title	Study Type
Dada 2022 [13]	Trabeculectomy Augmented with Limited Deep Sclerectomy and Cyclodialysis with Use of Scleral Tissue as a Spacer	Case Report
Dada 2021 [8]	Efficacy of Trabeculectomy Combined with Limited Deep Sclerectomy Versus Trabeculectomy Alone A Randomised-controlled Trial	Randomised Controlled Trial
Chan 2020 [17]	The Tenons’ Layer Reposition Approach of Trabeculectomy: A Longitudinal Case Series of a Mixed Group of Glaucoma Patients	Non-comparative case series
Olawoye 2015 [19]	Fornix-based Trabeculectomy with Mitomycin C Using the Horizontal Conjunctival Suture Technique	Non-comparative case series
Allam R 2020 [15]	Trabeculectomy With Extended Subscleral Tunnel Versus Conventional Trabeculectomy in the Management of POAG: A 1-Year Randomised-controlled Trial	Randomised Controlled Trial
Kirk 2014 [18]	Modified Wise Closure of the Conjunctival Fornix-Based Trabeculectomy Flap	Retrospective Comparative Study
Figus M 2016 [22]	Scleral Flap-Everting Suture for Glaucoma-Filtering Surgery	Non-comparative Case Series
Baykara M 2017 [23]	A Novel Suturing Technique for Filtering Glaucoma Surgery: The Accordion Suture	Non-comparative Case Series
**Glaucoma Drainage Device Studies**		
Grover 2022 [55]	Clinical Outcomes of Ahmed ClearPath Implantation in Glaucomatous Eyes: A Novel Valveless Glaucoma Drainage Device	Retrospective Case Series
Nakamura 2022 [120]	Tissue Reactivity to, and Stability of, Glaucoma Drainage Device Materials Placed Under Rabbit Conjunctiva	Animal In Vivo Study
Gupta 2021 [38]	A Graft-Free Scleral Sleeve Technique of Ahmed Glaucoma Valve Implantation In Refractory Glaucoma—Rising to the Challenge of COVID-19 Pandemic	Case Report
Gupta 2020 [30]	Pars Plana Placement of Ahmed Glaucoma Valve Tube Through Sclerotomy Port In Refractory Glaucoma: A Novel Surgical Technique	Case Report
Koh 2020 [53]	Treatment Outcomes Using the PAUL Glaucoma Implant to Control Intraocular Pressure in Eyes with Refractory Glaucoma	Interventional Cohort Study
Mungale 2019 [41]	A Novel Simplified Method for Managing Inadvertent Tube Cut During Aurolab Aqueous Drainage Implant Surgery For Refractory Glaucoma	Case Report
Roy 2019 [59]	Initial Clinical Results of the eyeWatch: A New Adjustable Glaucoma Drainage Device Used in Refractory Glaucoma Surgery	Prospective Non-comparative Clinical Trial
Sastre-Ibanez 2019 [40]	Efficacy of Ologen Matrix Implant in Ahmed Glaucoma Valve Implantation	Prospective Randomised Clinical Trial
Eslami 2019 [35]	Single Long Scleral Tunnel Technique for Prevention of Ahmed Valve Tube Exposure	Retrospective Case Series
Vergados 2019 [121]	Ab Interno Tube Ligation for Refractory Hypotony Following Non-valved Glaucoma Drainage Device Implantation	Retrospective Case Series
Pakravan 2018 [37]	Ahmed Glaucoma Valve Implantation: Graft-Free Short Tunnel Small Flap versus Scleral Patch Graft After 1-Year Follow-up: A Randomised Clinical Trial	Randomised Controlled Trial
Chiang 2017 [50]	A Novel Method of Extending Glaucoma Drainage Tube: “Tube-in-Tube” Technique	Retrospective Non-comparative Case Series
Hwang 2017 [42]	Intracameral Air Injection During Ahmed Glaucoma Valve Implantation In Neovascular Glaucoma for the Prevention of Tube Obstruction with Blood Clot: Case Report	Case Report
Brouzas 2017 [36]	Double Scleral Tunnel In Tandem Technique for Glaucoma Drainage Tube Implants	Case Series
Dervan 2017 [47]	Intermediate-Term and Long-Term Outcome of Piggyback Drainage: Connecting Glaucoma Drainage Device to a Device In Situ for Improved Intraocular Pressure Control	Retrospective Interventional Cohort Study
Park 2016 [122]	Polymeric Check Valve With an Elevated Pedestal for Precise Cracking Pressure In a Glaucoma Drainage Device	In Vitro Study
Kataria 2016 [43]	A Novel Technique of a Transcorneal Suture to Manage an Iris Tuck into the Tube of a Glaucoma Drainage Device	Case Report
Ahn 2016 [65]	Novel Membrane-Tube Type Glaucoma Shunt Device for Glaucoma Surgery	Retrospective Non-comparative Interventional Case Series
Gil-Carrasco 2016 [52]	Comparative Study of the Safety and Efficacy of The Ahmed Glaucoma Valve Model M4 (High-Density Po-Rous Polyethene) And the Model S2 (Polypropylene) In Patients With Neovascular Glaucoma	Prospective Comparative Randomised Study
Ma 2016 [34]	Modified Scleral Tunnel to Prevent Tube Exposure In Patients With Refractory Glaucoma	Retrospective Case Series
Maldonado-Junyent 2015 [32]	Oculo-Peritoneal Shunt: Draining Aqueous Humour To The Peritoneum	Case Report
Martino 2015 [123]	Surgical Outcomes of Superior Versus Inferior Glauco-Ma Drainage Device Implantation	Retrospective Case Series
Schaefer 2015 [44]	Failed Glaucoma Drainage Implant: Long-Term Out-Comes of a Second Glaucoma Drainage Device Versus Cyclophotocoagulation	Non-randomised Retrospective Cohort Study
Välimäki 2015 [46]	Insertion of Sequential Glaucoma Drainage Implant in a Piggyback Manner	Retrospective Case Series
Lee 2014 [45]	Efficacy of Additional Glaucoma Drainage Device Insertion in Refractory Glaucoma: Case Series with a Systematic Literature Review and Meta-Analysis	Non-comparative Retrospective Case Series
Luong 2014 [124]	A New Design and Application of Bioelastomers for Better Control of Intraocular Pressure In a Glaucoma Drainage Device	In Vitro Study
Grover 2013 [28]	Confirming and Establishing Patency of Glaucoma Drainage Devices Using Trypan Blue	Case Report
**Minimally Invasive Glaucoma Surgery Studies**		
Geffen 2022 [118]	Minimally Invasive Micro Sclerostomy (MIMS) Procedure: A Novel Glaucoma Filtration Procedure	Prospective Clinical Trial
Martinez-de-la-casa 2022 [81]	Posterior Chamber Implantation of a Preserflo Microshunt In a Patient With a Compromised Endothelium	Case Report
New World Medical 2022 [120]	STREAMLINE®SURGICAL SYSTEM Compared to iStent Inject W® in Patients with Open-Angle Glaucoma	Prospective Randomised Controlled Trial
Lin 2022 [87]	Accurate Identification of the Trabecular Meshwork Under Gonioscopic View in Real Time Using Deep Learning.	Cross-Sectional Study
Bleeker 2022 [95]	Short-Term Efficacy of Combined ab Interno Canaloplasty and Trabeculotomy in Pseudophakic Eyes with Open-Angle Glaucoma	Retrospective Case Series
Lazcano-Gomez 2022 [119]	Interim Analysis of STREAMLINE® Surgical System Clinical Outcomes in Eyes with Glaucoma	Prospective Case Series
Gallardo 2022 [77]	Comparison of Clinical Outcomes Following Gel Stent Implantation via Ab externo and Ab interno Approaches in Patients with Refractory Glaucoma.	Retrospective Case Series
Tan 2021 [75]	Comparison of Safety and Efficacy Between Ab Interno and Ab Externo Approaches to XEN Gel Stent Placement	Retrospective Case Series
Do 2021 [76]	Clinical Outcomes with Open Versus Closed Conjunctiva Implantation of the XEN45 Gel Stent	Retrospective Case Series
Feijoo 2020 [105]	A European Study of the Performance and Safety of MINIject in Patients with Medically Uncontrolled Open-angle Glaucoma (STAR-II)	Prospective Clinical Trial
Ucar 2020 [78]	Xen Implantation in Patients With Primary Open-Angle Glaucoma: Comparison of Two Different Techniques	Retrospective Comparative Interventional Study
Vera 2020 [79]	Surgical Approaches for Implanting Xen Gel Stent without Conjunctival Dissection	Expert Opinion
Ishida 2020 [85]	Observation of Gonio Structures during Microhook Ab Interno Trabeculotomy Using a Novel Digital Microscope with Integrated Intraoperative Optical Coherence Tomography	Retrospective Observational Study
Bravetti 2020 [99]	Xen-Augmented Baerveldt Drainage Device Implantation in Refractory Glaucoma: 1-Year Outcomes	Retrospective Case Series
Denis 2019 [100]	A First-in-Human Study of the Efficacy and Safety of MINIject in Patients with Medically Uncontrolled Open-Angle Glaucoma (STAR-I)	Randomised Controlled Trial
Laroche 2019 [109]	Intra-Scleral Ciliary Sulcus Suprachoroidal Microtube: Making Supraciliary Glaucoma Surgery Affordable	Case Report
Valimaki 2018 [46]	Xen Gel Stent to Resolve Late Hypotony After Glaucoma Drainage Implant Surgery: A Novel Technique	Case Report
Yen 2018 [51]	Pars Plana Insertion of Glaucoma Shunt in Eyes With Refractory Neovascular Glaucoma: Case Report	Case Report
Fili 2018 [104]	The Starflo Glaucoma Implant: Preliminary 12 Months Results	Prospective Case Series
